# Advancing e-commerce user purchase prediction: Integration of time-series attention with event-based timestamp encoding and Graph Neural Network-Enhanced user profiling

**DOI:** 10.1371/journal.pone.0299087

**Published:** 2024-04-18

**Authors:** Shuang Zhou, Norlaile Salleh Hudin

**Affiliations:** Sultan Idris Education University, Tanjung Malim, Perak, Malaysia; Harbin Institute of Technology, CHINA

## Abstract

In recent years, the global e-commerce landscape has witnessed rapid growth, with sales reaching a new peak in the past year and expected to rise further in the coming years. Amid this e-commerce boom, accurately predicting user purchase behavior has become crucial for commercial success. We introduce a novel framework integrating three innovative approaches to enhance the prediction model’s effectiveness. First, we integrate an event-based timestamp encoding within a time-series attention model, effectively capturing the dynamic and temporal aspects of user behavior. This aspect is often neglected in traditional user purchase prediction methods, leading to suboptimal accuracy. Second, we incorporate Graph Neural Networks (GNNs) to analyze user behavior. By modeling users and their actions as nodes and edges within a graph structure, we capture complex relationships and patterns in user behavior more effectively than current models, offering a nuanced and comprehensive analysis. Lastly, our framework transcends traditional learning strategies by implementing advanced meta-learning techniques. This enables the model to autonomously adjust learning parameters, including the learning rate, in response to new and evolving data environments, thereby significantly enhancing its adaptability and learning efficiency. Through extensive experiments on diverse real-world e-commerce datasets, our model demonstrates superior performance, particularly in accuracy and adaptability in large-scale data scenarios. This study not only overcomes the existing challenges in analyzing e-commerce user behavior but also sets a foundation for future exploration in this dynamic field. We believe our contributions provide significant insights and tools for e-commerce platforms to better understand and cater to their users, ultimately driving sales and improving user experiences.

## 1 Introduction

### 1.1 Background

The globalization of e-commerce has surged dramatically in recent years, especially in the past year, its influence and value have been greatly amplified. According to related statistical data, global e-commerce sales reached a staggering $4.28 trillion in 2020 [1], and this number is expected to rise further to $5.4 trillion by 2024 [1]. This impressive growth not only showcases the enormous potential of e-commerce but also reveals the significant challenges and issues underlying it.

In this e-commerce boom, predicting user purchasing behavior has become the key to business success. In fact, with the proliferation of social media and smart devices, consumer behavior data has become increasingly abundant and complex. For instance, a recent survey indicates that nearly 80% of online consumers browse or bookmark multiple times before making a purchasing decision [2]. This trend not only reveals that consumer purchasing behaviors are becoming more diverse and nuanced, but it also offers valuable data resources for businesses. However, accurately predicting user purchasing intent from this intricate behavior data, such as browsing, bookmarking, and adding to cart, poses a significant challenge.

While traditional machine learning methods can to some extent address this predictive task, their model capacity and limitations in handling high-dimensional, temporal data often fall short in prediction accuracy. Specifically, traditional machine learning methods struggle to capture the deep patterns and implicit relationships in user behavior data, leading directly to prediction inaccuracies. Moreover, with the advent of the big data era, there has been an explosive growth in user behavior data, making data processing and analysis increasingly challenging. Furthermore, user purchasing behaviors are influenced by numerous factors, including personal preferences, social influence, economic conditions, etc. The combined effect of these factors makes predicting purchasing behaviors exceptionally complex. In addition, the wide variety of products, significant price differences, and frequent promotions on e-commerce platforms all influence user purchasing decisions, adding to the prediction difficulty.

Therefore, in order to stand out in fierce market competition, researching how to accurately predict user purchasing behavior, and optimizing product recommendations and ad placements, has become a core focus for both e-commerce enterprises and academic researchers. By delving into user purchasing behaviors, businesses cannot only enhance sales efficiency but also provide consumers with more personalized and accurate shopping recommendations, achieving a win-win for both business performance and user experience.

### 1.2 Main contributions

This paper presents a novel approach to addressing the challenges in e-commerce prediction tasks, focusing on enhancing the accuracy of purchase intent prediction and adapting to the dynamic e-commerce environment. The key contributions are summarized in three innovative aspects:

- **Time-Series Attention Model with Event-Based Timestamp Encoding**: We innovate beyond traditional user purchase prediction methods by introducing a time-series attention model that incorporates event-based timestamp encoding. This approach captures the temporal dynamics in user actions more effectively, allowing for a nuanced understanding of user behavior and significantly improving the accuracy of predicting purchase behavior.
- **Graph Neural Network Enhanced User Profiling and Behavior Fusion**: To tackle the limitations of conventional user behavior analysis models, our framework integrates Graph Neural Networks (GNNs) for a deeper analysis of user behavior. By modeling users and their actions as a graph structure, the GNNs unravel complex relationships and patterns in user behavior, thereby enriching the understanding of the dynamic and interconnected nature of online user activities.
- **Enhanced Model Adaptability through Meta-Learning**: Our framework advances beyond traditional learning methods by integrating meta-learning techniques, enabling the model to autonomously adjust learning parameters in response to new data and domains. This innovative approach significantly boosts the model’s flexibility and learning efficiency, making it adept at handling rapidly changing data environments and diverse learning tasks.

## 2 Literature review

### 2.1 Time series analysis and forecasting

Time series analysis is a pivotal technique in statistics and data science, extensively employed in forecasting stock markets, weather, traffic flows, and more. This method focuses on predicting future values based on historical data. Particularly in the e-commerce domain, time series analysis has been used to forecast user purchasing behaviors and product sales volumes [3]. Traditional time series analysis methods, such as the ARIMA (AutoRegressive Integrated Moving Average) model and Exponential Smoothing methods, although capable, tend to underperform when dealing with complex, high-dimensional, and non-linear data [4].

Time series analysis is crucial in predicting user purchasing behaviors in e-commerce, where user actions exhibit pronounced temporal dependencies. Recent advancements in deep learning, such as the applications of RNN and LSTM, have transformed traditional approaches [5]. However, these models face challenges like computational intensity and inability to capture long-term dependencies effectively [6, 7]. Furthermore, after achieving massive success in the field of natural language processing, the attention mechanism began to be applied to time series analysis. This mechanism allows models to focus more on specific time points during predictions, enhancing forecasting accuracy [8]. In e-commerce, given the pronounced temporal dependencies in user behavior data, time series analysis is crucial. Additionally, the analysis must also consider individual user differences, product characteristics, seasonal factors, and more [9]. Thus, accurately and efficiently performing time series analysis to predict user purchasing behaviors remains a challenging and research-worthy problem.

In the e-commerce domain, Lara-Benítez [10] proposed a time series forecasting model based on deep neural networks. By incorporating additional features like users’ historical purchase records and price fluctuations of products, the model significantly improved prediction accuracy. However, it did not take into account product seasonality and promotional events, and the model’s performance significantly drops when faced with long-term dependencies. Chen [11], building on Lara-Benítez [10]’s work, introduced a multi-model fusion approach that considers seasonality and promotional events. While it addressed some of the previously mentioned issues to some extent, it still did not fully exploit the long-term dependency information in the time series. Pan [7] proposed a hybrid model based on LSTM and attention mechanisms to effectively capture both long-term and short-term dependencies in time series, substantially enhancing the accuracy of purchase predictions. However, his model demands significant computational resources, making it potentially unsuitable for real-time forecasting scenarios. These studies indicate that designing a model capable of handling complex time series dependencies while also catering to real-time prediction requirements remains a pressing issue.

The limitations of existing time series analysis methods, such as their inadequacy in handling complex, high-dimensional data and lack of adaptability in dynamic e-commerce environments, necessitate advanced analytical frameworks [4, 11].

### 2.2 User behavior analysis

E-commerce platforms such as Taobao and JD.com have a profound demand for user behavior analysis. This is not only because it can increase sales, but also because through precise analysis, platforms can effectively carry out advertising placements and personalized recommendations. User behavior analysis mainly focuses on different stages of actions like browsing, collecting, adding to the cart, and purchasing.

In the context of e-commerce, understanding and analyzing online user behavior, including aspects of habituation and decision-making processes, has become increasingly significant. Recent studies such as Sulikowski [12, 13] have emphasized the importance of visual aspects and layout in recommendation systems, which significantly impact user purchase decisions. Additionally, the work by Yildiz [14] introduces a hyper-personalized product recommendation system focused on customer segmentation, highlighting the evolving nature of e-commerce strategies.

Traditional user behavior analysis methods often revolve around using basic statistical models to depict user actions [15]. However, these methods tend to overlook the inherent connections and temporality between user behaviors [16, 17]. For instance, Kim [18] analyzed user purchasing behavior through basic statistical models, but it struggled to handle the intricate time dependencies between user actions. With the rise of deep learning, sophisticated models like Convolutional Neural Networks (CNN) and Recurrent Neural Networks (RNN) have been employed to capture intricate patterns in user behaviors [19]. Zhao [20]’s study, which utilized CNNs to identify patterns in user behaviors, achieved some success, but fell short in processing sequential data, thus limiting prediction accuracy.

In addition to traditional time-series analysis and deep learning techniques, novel approaches in purchase intent modeling have emerged in recent e-commerce studies. A fuzzy approach to model purchase intent, as proposed by Sulikowski [21], leverages user tracking data to create more personalized e-commerce recommenders. This method appreciates data uncertainty and provides insights into customer behavior in online shopping environments. Similarly, the analysis of unplanned purchase rules based on rough sets, as explored by Shibata [22], offers an understanding of consumer decision-making processes, particularly distinguishing between planned and unplanned purchases. These approaches underscore the importance of capturing nuanced buyer behaviors in the digital marketplace.

More recent studies have started integrating attention mechanisms into user behavior analysis. These methods can automatically assign different weights based on the significance of various behaviors, leading to more precise predictions [23]. Zhou [24]’s study attempted to employ the attention mechanism to weight various user interactions, hoping to more accurately predict purchase intentions. This is crucial as, during the shopping process, different types of actions (like collecting or adding to the cart) may have varying impacts on purchase intentions. However, his model is computationally inefficient when facing large-scale real-time data, making it hard to fulfill the needs of real-time recommendations.

There is a growing trend towards employing sophisticated analytical techniques to understand user behavior in e-commerce platforms, as evidenced by recent literature focusing on predictive and evaluative frameworks [12–14].

In summary, building a model that can both capture intricate patterns in user behavior and efficiently process vast amounts of data is crucial for enhancing e-commerce platform sales and user experience. After an in-depth analysis of previous studies, our research aims to address these challenges, proposing a novel framework for user behavior analysis based on deep learning and attention mechanisms. We hope to offer a fresh perspective and practical value to the e-commerce field.

### 2.3 Critical review of traditional learning methods and the emergence of meta-learning

In addressing the limitations of traditional learning strategies and enhancing our model’s performance, we turn to advanced meta-learning techniques. This approach is motivated by a critical review of recent literature, which highlights several shortcomings in existing methods.

Li [25] identified a significant limitation in traditional learning methods: the infeasibility of storing and retraining on all tasks as the number of tasks grows. This challenge necessitates more efficient learning strategies that can adapt without forgetting previous knowledge. Following this, Jan [26] pointed out the inadequacies of traditional data processing techniques in handling large volumes of data. This finding indicates a pressing need for robust learning frameworks capable of efficiently processing and learning from big data.

Teney [27] further highlighted the impracticality of traditional machine learning methods, which require training data to encompass all information to be learned. In dynamic and diverse data environments, this approach is unfeasible, suggesting a need for models that can adaptively learn from live data. In the same vein, Wang [28] underscored the limited effectiveness of traditional methods like cutout and random convolution in deep reinforcement learning, pointing to a gap in generalization performance that could be bridged by more adaptive learning strategies.

These two significant studies Xu [29] and Osa [30] collectively critiqued traditional ensemble learning methods and reinforcement learning algorithms for their lack of adaptability to noise, redundant features, and in learning diverse solutions. This underscores the necessity for more sophisticated, adaptable learning approaches.

Additionally, a series of papers between 2020 and 2022 focusing on e-commerce customer behavior prediction, including works by [16, 31, 32], highlighted the challenges faced by traditional machine learning approaches in e-commerce. These challenges include the need for more integrated and adaptive methods to accurately predict customer behavior.

The gap in traditional learning strategies, particularly in the context of e-commerce user behavior analysis, has been highlighted in recent research, underscoring the need for more integrated and adaptive methods [29, 30, 32]. Our work aims to bridge these gaps by introducing a novel framework that integrates time-series analysis, graph neural networks, and meta-learning techniques.

This study identifies key research gaps in the field of e-commerce user behavior analysis, particularly in the areas of dynamic behavior modeling, real-time data processing, and personalized recommendation accuracy. Our contribution lies in developing a comprehensive framework that addresses these challenges by integrating advanced time-series models, graph-based analysis, and meta-learning strategies. Our approach significantly enhances the prediction accuracy of user purchase behavior and offers practical insights for e-commerce platforms to optimize their strategies and user experiences.

In summary, our literature review establishes the context for our work by detailing the evolution of user behavior analysis in e-commerce, the challenges of existing predictive models, and the potential of advanced methodologies to offer more accurate and efficient solutions.

## 3 Our approach

### 3.1 Problem description

#### 3.1.1 Symbols and terminology

To describe the problem and methods systematically and clearly, we first define the main symbols and terms used in this paper.

- *U*: Set of users, representing all participants. *U* = {*u*_1_, *u*_2_, …, *u*_*N*_}, where *N* is the total number of users.
- *A*: Set of user actions, i.e., *A* = {Browsing, Favorites, Add to Cart, Purchase}.
-
$S_{u_{i}}$ : Action sequence of user *u*_*i*_, describing all actions of the user within the observation time window. $S_{u_{i}}=\left[\left(t_{1},a_{1}\right),\left(t_{2},a_{2}\right),\ldots ,\left(t_{n},a_{n}\right)\right]$.
- *t*_*j*_: Timestamp of event *a*_*j*_.
-
$C_{u_{i}}$ : Contextual information of user *u*_*i*_, which may include geographical location, device information, etc.
- *f*: Prediction function used to forecast based on the action sequence $S_{u_{i}}$ and contextual information $C_{u_{i}}$ whether the user will make a purchase.
- *L*(*f*): Loss function to evaluate the performance of prediction function *f*.

#### 3.1.2 Problem definition

This paper investigates how to use various user behaviors from online shopping datasets including browsing, favoriting, adding to cart, and purchasing—to predict whether a user will make a purchase. To systematically address this challenge, we first define some fundamental mathematical symbols and concepts.

Let *U* be the set of users, *U* = {*u*_1_, *u*_2_, …, *u*_*N*_}, where *N* is the number of users. Let *A* be the set of user actions, *A* = {*a*_1_, *a*_2_, …, *a*_*M*_}, where *M* is the type of action (in this paper, *M* = 4 corresponding to browsing, favoriting, adding to cart, and purchasing).

Each user *u*_*i*_ has an action sequence $S_{u_{i}}$, where $S_{u_{i}}=\left[\left(t_{1},a_{1}\right),\left(t_{2},a_{2}\right),\ldots ,\left(t_{n},a_{n}\right)\right]$. Each (*t*_*j*_, *a*_*j*_) represents the action *a*_*j*_ taken by user *u*_*i*_ at time *t*_*j*_.

Our goal is to construct a function *f*, which, based on the action sequence $S_{u_{i}}$ of user *u*_*i*_ and other potential contextual information $C_{u_{i}}$, predicts whether the user will make a purchase. Specifically:

$$\begin{array}{c} f:S_{u_{i}}\times C_{u_{i}}\to \left\{0,1\right\} \end{array}$$ (1)

where 0 indicates no purchase, and 1 indicates a purchase.

More specifically, we aim to minimize the following loss function:

$$\begin{array}{c} L\left(f\right)=\sum_{i=1}^{N}[y_{i}\text{log}\left(f\left(S_{u_{i}},C_{u_{i}}\right)\right)+\left(1-y_{i}\right)\text{log}\left(1-f\left(S_{u_{i}},C_{u_{i}}\right)\right)] \end{array}$$ (2)

where *y*_*i*_ represents the actual purchasing behavior of user *u*_*i*_ (either 0 or 1).

### 3.2 Motivation for time-series attention model with event-based timestamp encoding

- **Limitations of Current Methods**: Traditional methods in user purchase prediction primarily focus on static features such as demographic information and historical purchase records [33]. These approaches often fail to effectively capture the dynamic and temporal aspects of user behavior [31, 34], such as the timing of actions like browsing, adding to cart, or saving items, which are critical for understanding and predicting user purchasing decisions.
- **Our Innovation—Event-Based Timestamp Encoding**: To address these limitations, we introduce an innovative time-series attention model that incorporates event-based timestamp encoding. This approach allows for a more nuanced capture of the temporal dynamics in user actions, significantly enhancing the model’s ability to predict purchase behavior with higher accuracy. By integrating the precise timing of user interactions, our model can identify patterns and trends that traditional models overlook.

### 3.3 Time-series attention model with event-based timestamp encoding

Building on the motivations discussed above, this section details the mathematical structure of the time-series attention model, now enhanced with an event-based timestamp encoding mechanism.

#### 3.3.1 Mathematical definition

**Definition 1**. *A **time-series attention model with event-based timestamp encoding** is a function* $f:R^{T\times D}\to R$, *incorporating event timestamps into the traditional time-series model. The function can be modified as*:

$$\begin{array}{c} f\left(X,E\right)=Softmax(\sum_{t=1}^{T}\alpha_{t}h_{t}) \end{array}$$ (3)

*where α*_*t*_ *are the attention weights, incorporating the event-based timestamp encoding* **E**, *calculated as*:

$$\begin{array}{c} \alpha_{t}=\frac{\text{exp}(Attention\left(h_{t},C,E_{t}\right))}{\sum_{j=1}^{T}\text{exp}\left(Attention\left(h_{j},C,E_{j}\right)\right)} \end{array}$$ (4)

**h**_*t*_ *is the hidden state at time step t*, **C** *is the context vector, and* **E**_*t*_ *is the event-based timestamp at time step t*.

**Definition 2**. *The **Attention function** is modified to include the event timestamp*:

$$\begin{array}{c} Attention\left(h_{t},C,E_{t}\right)=h_{t}^{T}WC+Encode\left(E_{t}\right)+b \end{array}$$ (5)

*where* **W** *is the learnable weight matrix*, **b** *is the bias vector, and Encode*(**E**_*t*_) *is the encoding function for the event timestamp*.

#### 3.3.2 Optimization with event-based timestamp encoding

The integration of event-based timestamp encoding into our time-series attention model leads to a more complex and nuanced optimization problem. We seek to minimize a loss function that not only considers the traditional features but also the temporal dynamics of user actions.

$$\begin{array}{c} L\left(\Theta \right)=-\sum_{i=1}^{N}(y_{i}\text{log}\left(f\left(X_{i},E_{i}\right)\right)+\left(1-y_{i}\right)\text{log}\left(1-f\left(X_{i},E_{i}\right)\right))+{\lambda ||\Theta ||}_{2}^{2} \end{array}$$ (6)

where Θ represents the model parameters, *N* is the number of samples, *y*_*i*_ is the true label of the *i* − *th* sample, **X**_*i*_ is the feature matrix, and **E**_*i*_ is the event-based timestamp matrix for the *i* − *th* sample.

Incorporating the event-based timestamps requires modifying the traditional attention mechanism. The attention weights, *α*_*t*_, are now a function of both the hidden state and the event timestamps:

$$\begin{array}{c} \alpha_{t}\left(\Theta \right)=\frac{\text{exp}(\text{Attention}(h_{t},C,E_{t};\Theta ))}{\sum_{j=1}^{T}\text{exp}(\text{Attention}(h_{j},C,E_{j};\Theta ))} \end{array}$$ (7)

where **h**_*t*_ is the hidden state at time step *t*, **C** is the context vector, **E**_*t*_ is the event-based timestamp at time step *t*, and Θ includes parameters specific to the event-based encoding.

The attention function itself is adapted to include the influence of the event timestamps:

$$\begin{array}{c} \text{Attention}\left(h_{t},C,E_{t};\Theta \right)=h_{t}^{T}W_{h}C+E_{t}^{T}W_{e}C+b \end{array}$$ (8)

where **W**_*h*_ and **W**_*e*_ are learnable weight matrices for the hidden states and event timestamps, respectively, and **b** is the bias vector.

This formulation allows our model to dynamically adjust the attention mechanism based on the specific timing of user actions, offering a more accurate and robust prediction model for user purchase behavior.

From above we derive the following theorem, whose proof process is shown in the S1 Appendix.

**Theorem 1**. ***Complex Optimization of Time-Series Attention Model**: Considering the time-series attention model with event-based timestamp encoding, defined as function* $f:R^{T\times D}\to R$, *the optimization of attention weights α*_*t*_ *can be expressed by a complex formulation*:

$$\begin{array}{c} \underset{\Theta }{\text{min}}L\left(\Theta \right)=-\sum_{i=1}^{N}\sum_{t=1}^{T}\alpha_{t,i}\cdot (y_{i,t}\text{log}\left(f\left(X_{i},E_{i};\Theta \right)\right)+\left(1-y_{i,t}\right)\text{log}\left(1-f\left(X_{i},E_{i};\Theta \right)\right))+{\lambda ||\Theta ||}_{2}^{2} \end{array}$$ (9)

*with α*_*t*,*i*_ *being calculated by*:

$$\begin{array}{c} \alpha_{t,i}=\frac{\text{exp}(Attention(h_{t,i},C,E_{t,i};\Theta ))}{\sum_{j=1}^{T}\text{exp}(Attention(h_{j,i},C,E_{j,i};\Theta ))} \end{array}$$ (10)

*where* **h**_*t*,*i*_ *is the hidden state at time step t for sample i*, *and* **C**, **E**_*t*,*i*_, Θ *represent the context vector, event-based timestamp, and model parameters respectively*.

### 3.4 Motivation for Graph Neural Network enhanced user profiling and behavior fusion

- **Limitations of Current Methods**: Traditional user behavior analysis models, including the time series attention model, primarily focus on temporal data, often neglecting the intricate interplay of user interactions and behaviors. These models lack the ability to capture complex relationships and patterns in user behavior, leading to a limited understanding of the dynamic and interconnected nature of online user activities [35, 36].
- **Our Innovation—Graph Neural Network-Based Analysis**: To overcome these limitations, we propose an innovative integration of Graph Neural Networks (GNNs) for analyzing user behavior. By modeling users and their actions as a graph structure, where nodes represent users and behaviors, and edges symbolize the relationships or sequences of actions, GNNs enable us to uncover deeper and more complex relationships in user behavior. This approach significantly enhances the model’s capability to analyze user behavior in a comprehensive and nuanced manner.

### 3.5 Graph Neural Network Enhanced user profiling and behavior fusion

Building on the foundation of user profiling and behavior fusion, we introduce a Graph Neural Network-based approach to further enhance our model’s predictive capabilities.

#### 3.5.1 Graph structure formulation

**Definition 3 (User-Behavior Graph)** *Let’s define a graph G* = (*V*, *E*), *where V represents the set of nodes, each node corresponding to a user or a specific user action, and E represents the set of edges, indicating the relationships or sequences of actions between users*.

#### 3.5.2 GNN-based user behavior analysis

**Definition 4 (Graph Neural Network Function)**. *A Graph Neural Network function* $\text{GNN}:R^{\left|V\right|}\times R^{\left|E\right|}\to R$ *is defined to process the graph structure, capturing the complex interrelations among users and their behaviors*.

$$\begin{array}{c} \text{GNN}\left(G\right)=\text{AGGREGATE}(\{\text{UPDATE}(v)\;|\;v\in V\}) \end{array}$$ (11)

where UPDATE(*v*) updates node representations based on their neighbors, and AGGREGATE combines these representations to capture the overall graph structure.

#### 3.5.3 Optimization with GNN-Based user behavior analysis

Integrating the Graph Neural Network (GNN) based analysis with the time series attention model requires advanced mathematical formulations, including calculus and linear algebra transformations. The fusion process is elaborated below:

Let *G* = (*V*, *E*) be the user-behavior graph with *V* as nodes (users and behaviors) and *E* as edges (relationships). The GNN function, GNN(*G*), captures complex interactions within this graph:

$$\begin{array}{c} \text{GNN}\left(G\right)=\text{AGGREGATE}(\{\text{TRANSFORM}(v)\;|\;v\in V\}) \end{array}$$ (12)

where TRANSFORM(*v*) updates the representation of each node incorporating its neighbors, and AGGREGATE merges these representations.

The transformation function for each node *v* is expressed as:

$$\begin{array}{cc} \text{TRANSFORM}(v) & =\text{ReLU}(W_{v}\cdot (\sum_{u\in \text{Neighbors}(v)}\text{RELATION}\left(u,v\right)))\\ & =\text{ReLU}(W_{v}\cdot (\int_{u\in \text{Neighbors}(v)}\text{RELATION}\left(u,v\right)\;du))\\ & =\text{ReLU}(W_{v}\cdot (\int A_{uv}{\cdot \text{exp}(-||u-v||}^{2})\;du)) \end{array}$$ (13)

where *W*_*v*_ is the weight matrix for node *v*, RELATION(*u*, *v*) models the relationship as a function of distance in feature space, and **A**_*uv*_ is the adjacency matrix.

The fusion of GNN output with the time series attention model output, *F*(*U*, *A*), is performed using a complex matrix operation:

$$\begin{array}{cc} F^{\prime }\left(U,A,G\right) & =F(U,A)⊕\text{GNN}(G)\\ & =(\sum_{i=1}^{m}\alpha_{i}a_{i})⊕(\sum_{v\in V}\text{TRANSFORM}\left(v\right))\\ & =(\sum_{i=1}^{m}\frac{\text{exp}(a_{i}^{T}W_{a})}{\sum_{j=1}^{m}\text{exp}\left(a_{j}^{T}W_{a}\right)}a_{i})⊕(\sum_{v\in V}\text{ReLU}(W_{v}\cdot \int A_{uv}{\cdot \text{exp}(-||u-v||}^{2})\;du)), \end{array}$$ (14)

where **W**_*a*_ and **W**_*v*_ are the weight matrices for the attention model and GNN respectively.

The final prediction model combines user profiles, behavior sequences, and graph-based relationships:

$$\begin{array}{c} \hat{y}=\sigma (F^{\prime }\left(U,A,G\right))=\sigma ((\sum_{i=1}^{m}\alpha_{i}a_{i})⊕(\sum_{v\in V}\text{TRANSFORM}\left(v\right))) \end{array}$$ (15)

where *σ* is the sigmoid activation function.

This fusion model, enhanced with complex mathematical operations, effectively combines temporal insights and intricate relational data, offering a highly advanced predictive model for user behavior.

From above we derive the following theorem and lemma, whose proof process is shown in the S1 Appendix.

**Theorem 2**. ***Solution Properties of Graph Neural Network Enhanced User Profiling**: Given the Graph Neural Network (GNN) formulation in* Eq (11), the solution of the GNN-based user profiling satisfies the following properties:

$$\begin{array}{cc} \forall v\in V,\;\exists h_{v}^{\prime }\in R^{D}\\ \text{s.t.}\; & \text{TRANSFORM}\left(v\right)=\text{ReLU}(W_{v}\cdot (\sum_{u\in \text{Neighbors}(v)}\text{RELATION}\left(u,v\right)))\\ & \text{And}\;\text{AGGREGATE}(\{h_{v}^{\prime }\;|\;v\in V\})\;\text{is}\;\text{bounded} \end{array}$$ (16)

**Lemma 1**. ***Convergence of GNN-Based User Behavior Analysis**: The Graph Neural Network (GNN) approach for user behavior analysis, as described by* Eq (11), *converges to a stable solution after a finite number of iterations*.

### 3.6 Motivation for enhancing model adaptability through meta-learning

To address the limitations of traditional learning strategies and elevate our model’s performance, we integrate advanced meta-learning techniques. This section discusses the shortcomings of existing methods and our innovative approach.

- **Limitations of Traditional Learning Methods**: Existing real-time update and transfer learning strategies predominantly rely on classical algorithms like Stochastic Gradient Descent (SGD). These methods, while effective in certain scenarios, lack the dynamic adaptability required to handle rapidly changing data environments and diverse learning tasks efficiently [37–39].
- **Our Innovation—Advanced Adaptive Learning Strategies**: To overcome these limitations, we propose integrating meta-learning methods into our model. Meta-learning, or learning to learn, allows the model to autonomously adjust its learning rate and other critical parameters, enhancing its ability to swiftly adapt to new data and domains. This approach not only improves the overall performance but also significantly boosts the model’s flexibility and learning efficiency.

### 3.7 Implementation of meta-learning, transfer learning, and advanced adaptation

Meta-learning introduces a higher level of adaptability and efficiency to the model’s learning process. It enables automatic adjustment of learning parameters based on incoming data, optimizing the model’s response to new information. We formalize this through the following mathematical framework:

**Definition 5 (Meta-Learning for Model Adaptation)**. *In meta-learning, the model parameters θ*_*t*_ *are updated not only based on the current data point* (*x*_*t*_, *y*_*t*_) *but also considering the learned strategy from historical data*.

The enhanced model adaptation using meta-learning is described by:

$$\begin{array}{cc} \theta_{t+1} & =\theta_{t}-\eta_{t}\nabla L\left(\theta_{t},x_{t},y_{t}\right)\\ \eta_{t} & =\Phi (H_{t}M) \end{array}$$ (17)

where *η*_*t*_ is the adaptive learning rate determined by the meta-learning function Φ, $H_{t}$ represents the historical data, and M denotes the meta-parameters.

The optimization of the meta-learning function Φ is crucial for the adaptability and efficiency of our model. This process involves complex mathematical formulations, such as the use of Lagrange multipliers for constraint optimization. We detail this process as follows:

$$\begin{array}{cc} \underset{M}{\text{min}}\; & \sum_{t=1}^{T}L\left(\theta_{t},x_{t},y_{t}\right)+\lambda R\left(M\right)\\ \text{s.t.}\; & \theta_{t+1}=\theta_{t}-\Phi \left(H_{t},M\right)\nabla L\left(\theta_{t},x_{t},y_{t}\right) \end{array}$$ (18)

Here, R(M) is a regularization term for the meta-parameters with λ as the regularization coefficient. To integrate these constraints, we apply the method of Lagrange multipliers:

$$\begin{array}{cc} L(M,\alpha ) & =\sum_{t=1}^{T}L\left(\theta_{t},x_{t},y_{t}\right)+\lambda R\left(M\right)+\sum_{t=1}^{T}\alpha_{t}(\theta_{t+1}-\theta_{t}+\Phi \left(H_{t},M\right)\nabla L\left(\theta_{t},x_{t},y_{t}\right))\\ & =\sum_{t=1}^{T}(L\left(\theta_{t},x_{t},y_{t}\right)+\alpha_{t}(\theta_{t+1}-\theta_{t}))+\lambda R\left(M\right)-\sum_{t=1}^{T}\alpha_{t}\Phi \left(H_{t},M\right)\nabla L\left(\theta_{t},x_{t},y_{t}\right) \end{array}$$ (19)

In this equation, *α* = (*α*_1_, *α*_2_, …, *α*_*T*_) represents the Lagrange multipliers. By setting the derivative of L with respect to *α*_*t*_ to zero, we obtain the conditions for optimal Φ:

$$\begin{array}{cc} \frac{\partial L}{\partial \alpha_{t}} & =\theta_{t+1}-\theta_{t}+\Phi \left(H_{t},M\right)\nabla L\left(\theta_{t},x_{t},y_{t}\right)=0\\ \Phi (H_{t},M) & =-(\theta_{t+1}-\theta_{t})/\nabla L\left(\theta_{t},x_{t},y_{t}\right) \end{array}$$ (20)

The decomposition of the meta-learning function Φ is:

$$\begin{array}{cc} \Phi (H_{t},M) & =\Psi (H_{t};W,B),\\ & =\sigma (W\cdot \text{Enc}(H_{t})+B)\\ & =\sigma (W\cdot (\sum_{i=1}^{n}h_{t,i}x_{i})+B) \end{array}$$ (21)

In this formulation, *Ψ* is a neural network with weights W and biases B, Enc is an encoder function for historical data, and *σ* is an activation function. The encoder function aggregates the historical data $H_{t}$ into a feature representation, which is then transformed by *Ψ*. The optimization of meta-parameters M={W,B} involves a complex process of minimizing the cumulative loss while adapting the learning strategy based on the historical data. This process ensures that the model learns the most effective strategy for parameter adaptation over time.

From above we derive the following lemma, whose proof process is shown in the S1 Appendix.

**Lemma 2**. ***Optimality of Meta-Learning Adaptation**: In the context of the meta-learning framework outlined in* Eqs (17), (18), (19) and (20), *the adapted model parameters θ*_*t*_ converge to an optimal set of parameters θ* *that minimize the cumulative loss over the training dataset while ensuring adaptability to new data*.

Through this advanced meta-learning implementation, incorporating complex mathematical optimization techniques, our model achieves superior adaptability and learning efficiency, making it highly suitable for dynamic and evolving data environments.

## 4 Algorithm pseudocode

In this section, we introduce the algorithmic implementation of the time series attention model. Algorithm 1 captures the importance of different time steps through an attention mechanism, as guided by Eqs (6), (7) and (8).

**Algorithm 1:** Time Series Attention Model Algorithm

**Input:** Time series dataset $D={\left\{\left(x_{t},y_{t}\right)\right\}}_{t=1}^{T}$, learning rate *η*, regularization parameter λ

**Output:** Attention weights *α*_*t*_ and model parameters Θ

**1** Initialize model parameters Θ to random values

**2** **for** *each epoch* **do**

**3**  Shuffle the dataset D;

**4**  **for** each mini-batch *B* in D **do**

**5**   **for** *each time step t in sequence* **do**

**6**    Compute intermediate representation *h*_*t*_;

**7**    Compute attention weights *α*_*t*_ using Eq (7);

**8**   **end**

**9**   Compute loss L using Eq (6);

**10**   Compute gradient *g*_*t*_ using Eq (8);

**11**   Update model parameters: Θ ← Θ − *η* ⋅ (*g*_*t*_ + λ ⋅ ∇*R*(Θ));

**12**  **end**

**13**  Evaluate the model on the validation set and apply early stopping if necessary;

**14**  **end**

**15** **return** *α*_*t*_, Θ;

Algorithm 2 focuses on combining user profiles with user behavior data, aligning with the concepts discussed in Eqs (17), (18), (19), (20) and (21).

**Algorithm 2:** User Profile and Behavior Fusion Algorithm

**Input:** User profile dataset $P={\left\{p_{i}\right\}}_{i=1}^{M}$, User behavior dataset $B={\left\{b_{j}\right\}}_{j=1}^{N}$, Time series dataset $D={\left\{\left(x_{t},y_{t}\right)\right\}}_{t=1}^{T}$, Learning rate *η*, Regularization parameter λ, Attention weights *α*_*t*_ and model parameters Θ (from Algorithm 1)

**Output:** Integrated user representation *R*_*u*_

**1** Initialize model parameters Φ to random values

**2 for** *each epoch* **do**

**3**  Shuffle the dataset D;

**4**  **for** each mini-batch *B* in D **do**

**5**   **for** each user *u* in mini-batch *B* **do**

**6**    Retrieve user *u*’s profile *p*_*u*_ from P;

**7**    Retrieve user *u*’s behavior *b*_*u*_ from B;

**8**    **for** *each time step t in sequence* **do**

**9**     Compute attention-augmented user behavior representation: *r*_*t*_ = *f*(*b*_*u*_, *x*_*t*_, *α*_*t*_, Θ) using Eqs (17) and (20);

**10**    **end**

**11**    Compute intermediate representation *h*_*u*_ = *g*(*p*_*u*_, *r*_*t*_, Φ) using Eqs. (19) and (21);

**12**   **end**

**13**   Compute loss L using Eq (18);

**14**   Compute gradient *g*_Φ_ using Eq (21);

**15**   Update model parameters: Φ ← Φ − *η* ⋅ (*g*_Φ_ + λ ⋅ ∇*R*(Φ));

**16**  **end**

**17**  Evaluate the model on validation set and apply early stopping if necessary;

**18** **end**

**19 return** *R*_*u*_ = *h*_*u*_;

## 5 Experiments

### 5.1 Dataset introduction

This study employs datasets on e-commerce user behavior analysis and live streaming e-commerce. These datasets offer valuable insights to e-commerce platforms, helping them to better understand consumers’ shopping habits, preferences, and motivations. Leveraging this data, e-commerce platforms can devise strategies to enhance conversion rates, boost customer loyalty, and optimize operational results. Below is a detailed introduction of these two datasets:

**E-commerce User Behavior Analysis Dataset**: This dataset primarily focuses on users’ online shopping behavior and their interactions with the website. By analyzing user features, merchants can identify key target user groups. Through website optimization, and understanding users’ behaviors on different pages, merchants can discern which pages are most popular and which may have issues or need optimization, thereby refining marketing strategies and enhancing conversion rates (available at https://tianchi.aliyun.com/dataset/124814).

**Live Streaming E-commerce Dataset**: This dataset centers on users’ interactions with live streaming shopping and their purchase behaviors. By analyzing user-related variables, merchants can predict user behaviors and adopt measures to devise more effective strategies to retain them (available at https://tianchi.aliyun.com/dataset/154063).

The main utility of these datasets lies in providing in-depth insights into e-commerce user behaviors. Through this data, e-commerce platforms can formulate more precise market strategies, optimize user experiences, increase conversion rates, and ultimately drive sales growth. Whether it’s traditional e-commerce or live streaming e-commerce, understanding the real needs and behaviors of users is paramount. Our experimental setup is shown in Table 1.

**Table 1:** E-Commerce user purchase prediction experiment parameters.

Parameter	Value	Parameter	Value
Dataset Size	50,000 User Sessions	Session Duration	1–60 minutes
Graph Nodes	75,000	Graph Edges	150,000
Time-Series Window	15 seconds	Frame Extraction Rate	1 frame/sec
Positive Sample Ratio	30%	Negative Sample Ratio	70%
GNN Layers	3 layers	LSTM Layers	2 layers
Filters per CNN Layer	128	Activation Function	ReLU
Meta-Learning Rate Adjust	Dynamic	Learning Rate	0.0005
Optimizer	Adam	Loss Function	Binary Cross-Entropy
Batch Size	64	Training Epochs	100
Validation Set Ratio	20%	Early Stopping	5 Epochs
Regularization Method	L2 Regularization	L2 Rate	0.02
Data Augmentation	Random Cropping, Flipping	Cropping Range	0.8–1.0
Event Timestamp Encoding	Customized	Attention Model	Moderate
Input Data Format	Mixed (Num., Cat.)	User Profile Features	Demographics, History
Evaluation Metrics	Accuracy, Precision, Recall	Test Set Ratio	20%
Data Preprocessing	Normalization, Encoding	Normalization Range	0–1
Experiment Environment	Cloud-based GPUs	GPUs Used	NVIDIA Tesla T4

### 5.2 Beginning the shopping journey–the collection phase

When users develop an interest in certain products, they might further inspect the product details and save them to their collection. This action indicates a user’s interest in a product; they might be comparing different products or contemplating a purchase. Data suggests that users browsing products are more likely to add them to their collection. The relationship between users’ browsing and collecting behaviors is pivotal for e-commerce platforms since a user’s collecting behavior might be an indicator of purchase intent.

**Fig 1:**
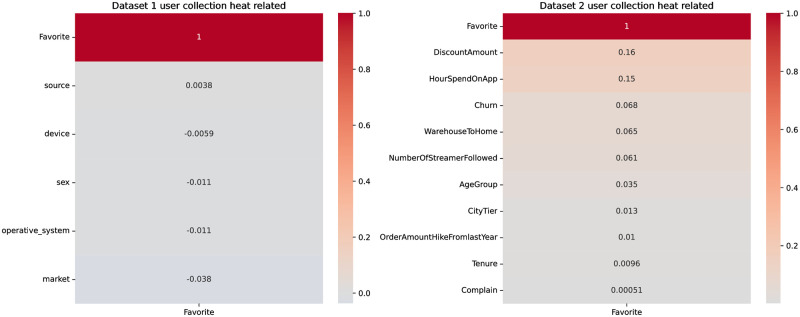
User collection heat related.

Heat correlation maps are data visualization methods used to display the correlation or association strength between influencing factors. Typically, heat correlation maps use colors to depict the degree of correlation between factors. Darker colors usually represent a perfect positive correlation, often denoted by 1, while colors that deviate from this signify less correlation, often denoted by lighter shades. We utilize the heat correlation map to explore the relationship between user collection behaviors and whether they collect, and factors that might influence these collection behaviors. As shown in Fig 1, we will subsequently analyze the top three factors influencing user collection behaviors and their relationship with the collection action.

Table 2 provides a detailed display of the collection behavior of users in Dataset 1. The activity of female users in shopping significantly surpasses that of male users. Direct access is the primary source, followed by traffic from search engines. Although traffic generated by advertisements is the least, it still plays a certain role. Mobile device visits evidently exceed those from desktop devices.

**Table 2:** Dataset 1: The relationship between whether a user collects it or not.

	Sex(Female)	Sex(Male)	Source(Direct)	Source(Seo)	Source(Ads)	Device(mobile)	Device(desktop)
Yes	44745	28235	41618	20563	10799	44270	28710
No	15498	10279	14459	7451	3867	15456	10321

**Fig 2:**
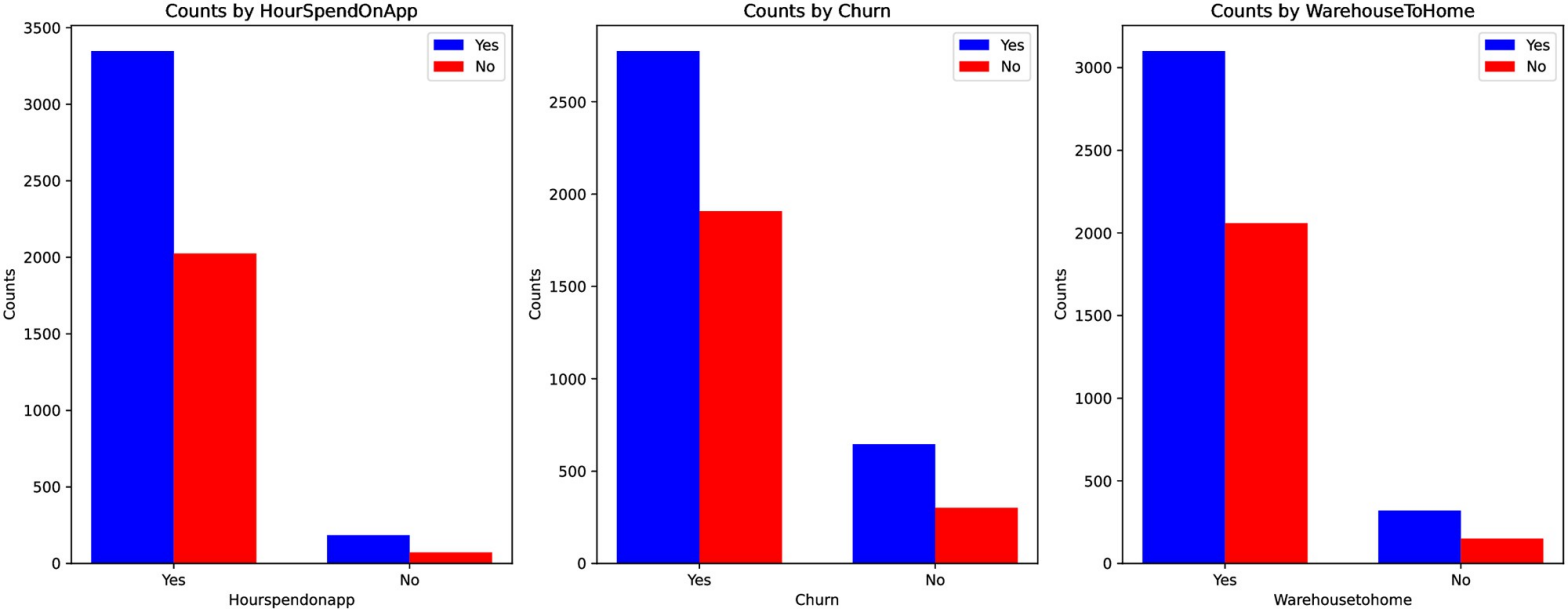
Dataset 2: The relationship between whether a user collects it or not.

Fig 2 showcases the collection behavior of users in Dataset 2. It can be observed that users who are willing to spend more time on products are more inclined to add them to their collections. Among users who collect, many continue to collect over a period of time. Products shipped from nearby locations are more likely to be collected.

**Fig 3:**
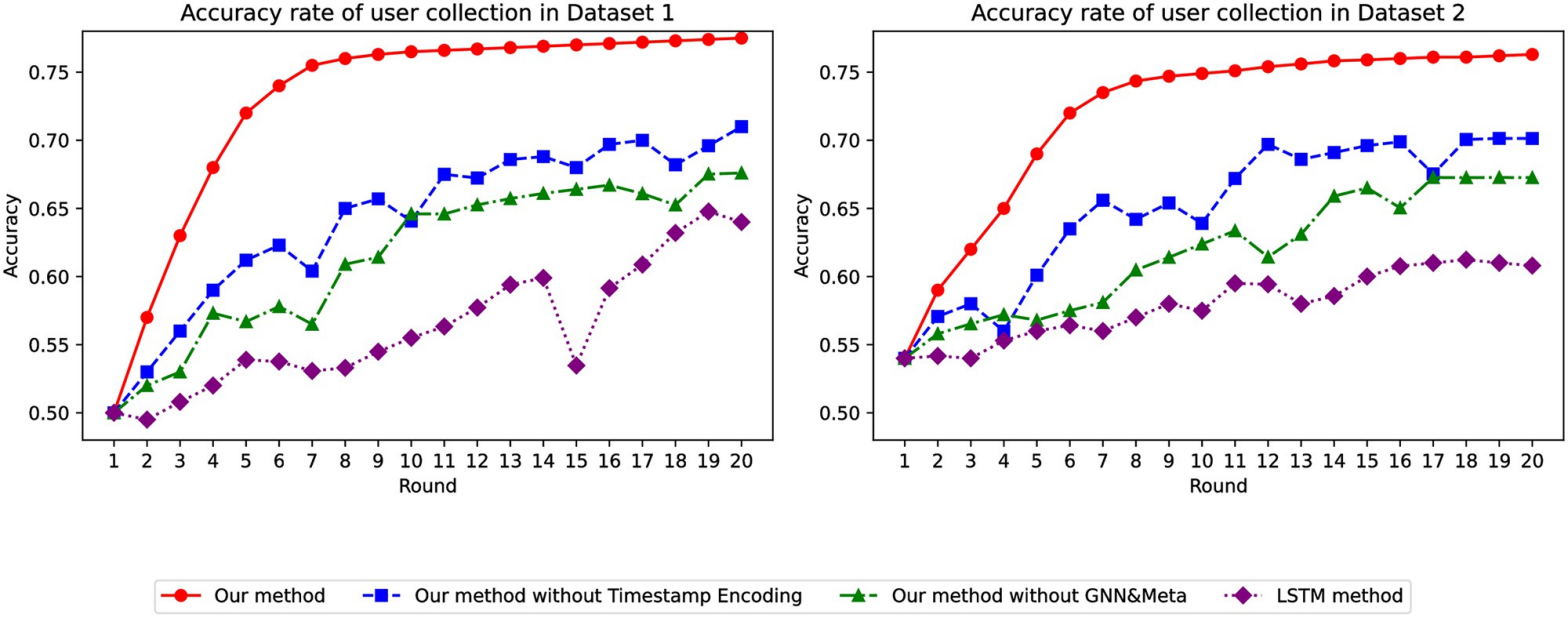
Accuracy of whether the user has collected it or not.

Through the comparison of various models, as shown in Fig 3, as the training epochs of the global model increase, the accuracy of our model steadily improves and is the highest. Additionally, our model’s accuracy on Dataset 1 is approximately 15% higher than that of traditional models. When using Dataset 2, the accuracy is also about 18% higher than traditional models. Moreover, during experimentation, we observed that traditional models are highly unstable, whereas our model performs remarkably well.

**Fig 4:**
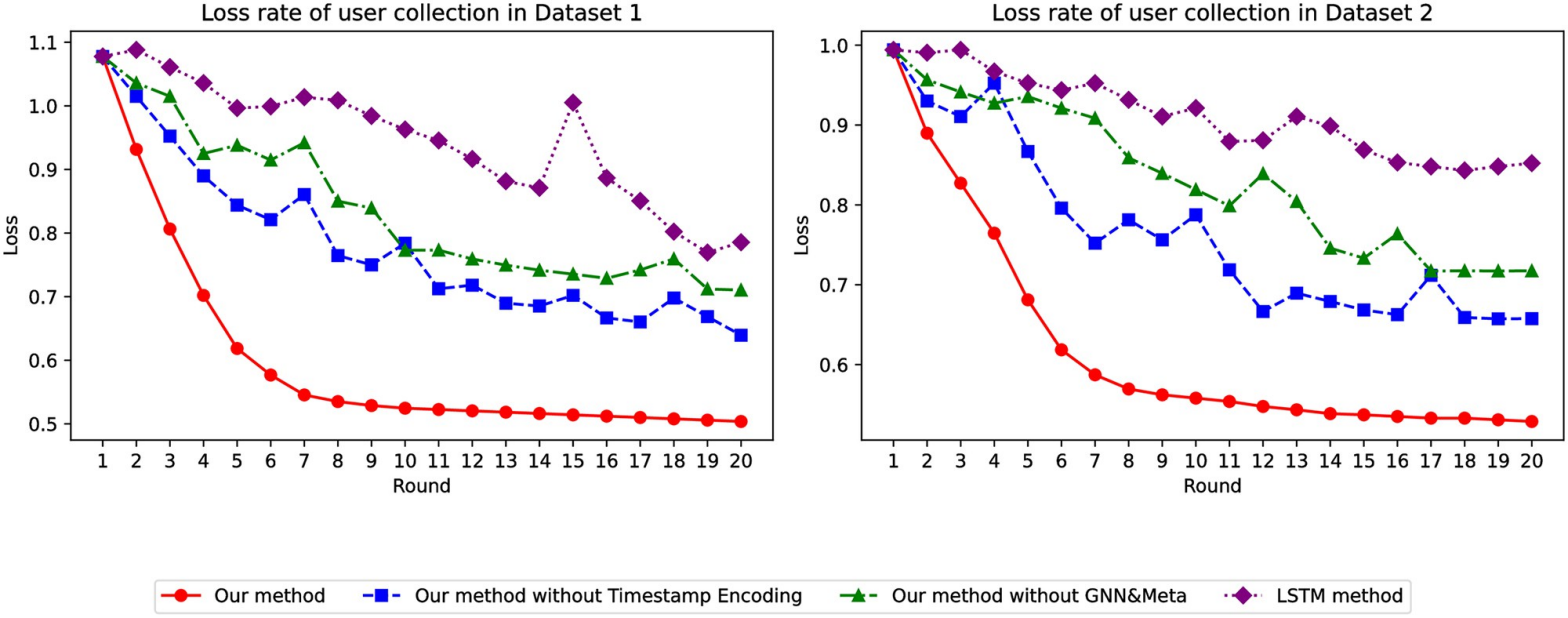
Loss of whether the user has collected it or not.

Regarding loss values, the stability of our model compared to traditional models is influenced by the training of the global model. As seen in Fig 4, as the number of epochs for both datasets increases, the loss value of the traditional model becomes more unstable, whereas our model achieves the smallest loss value, and only our model demonstrates relative stability and optimal performance.

**Fig 5:**
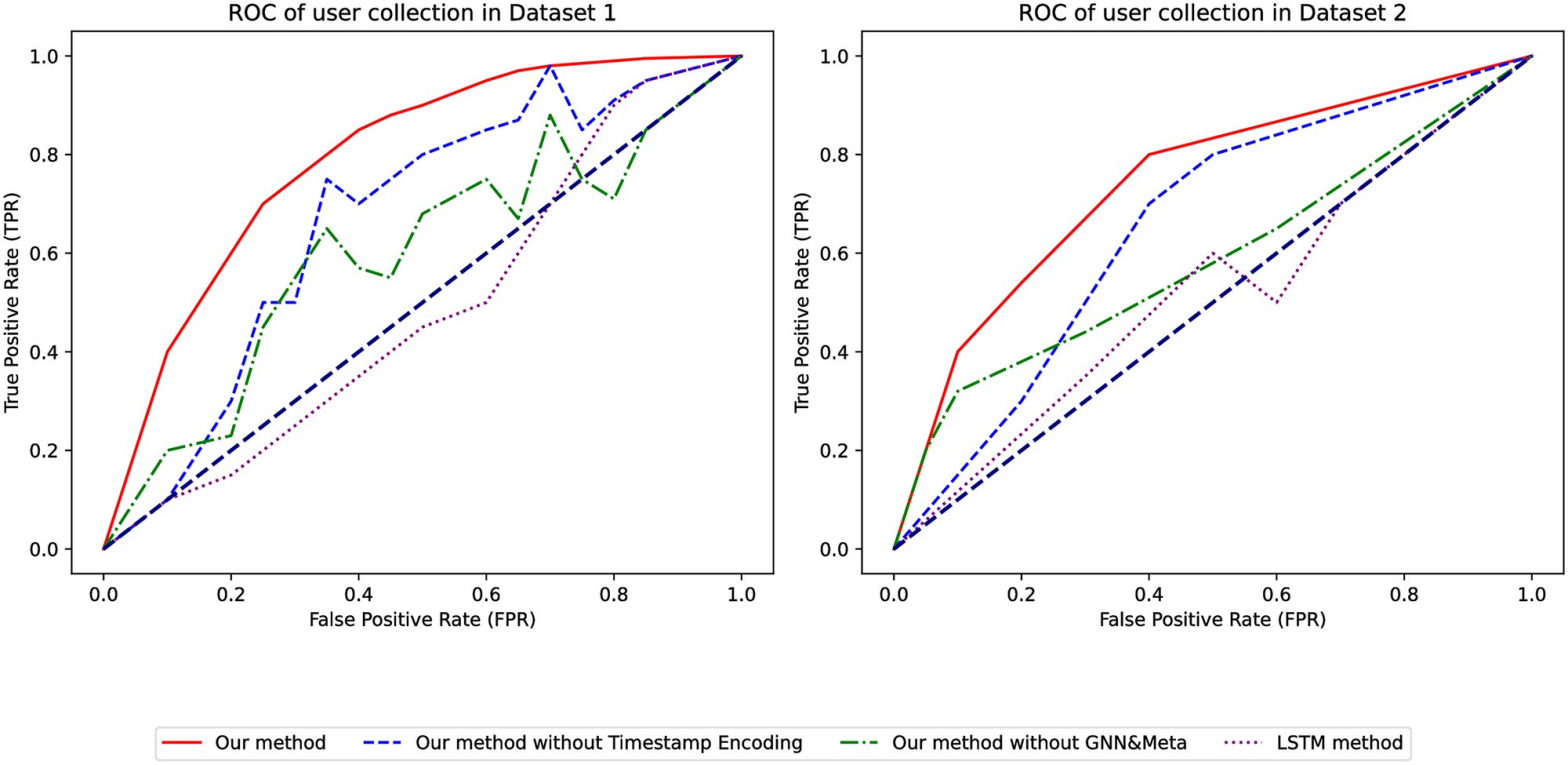
ROC of whether the user has collected it or not.

By comparing the ROC curves of the models, as depicted in Fig 5, the area under our model’s curve remains the largest and is above the dashed line in both datasets, indicating its superior performance over traditional models. Based on these comparisons, our model performs excellently and is far superior to traditional models.

For operators, they should pay more attention to female users, continuously optimize the content of product pages, strengthen search engine optimization, and ensure a good user experience on mobile devices. Offering more discounts or promotional events to attract users to collect, optimizing the user experience of the application, and improving the conversion rate of adding to the cart are essential. At the same time, using our model to predict user behavior can accurately recommend products to users within a certain traffic range on the platform.

### 5.3 Clear purchase intention–adding to cart

As users’ buying intentions become clearer, they might add products to their shopping cart. At this stage, users are very close to making a purchase decision. They might be considering the price, quality, delivery options, and other policies of the product. Adding to the cart is a clear signal that users are very interested in the product and are considering purchasing it in the near future. At this stage, e-commerce platforms can further stimulate users’ purchasing intentions by providing specific discounts or coupons.

**Fig 6:**
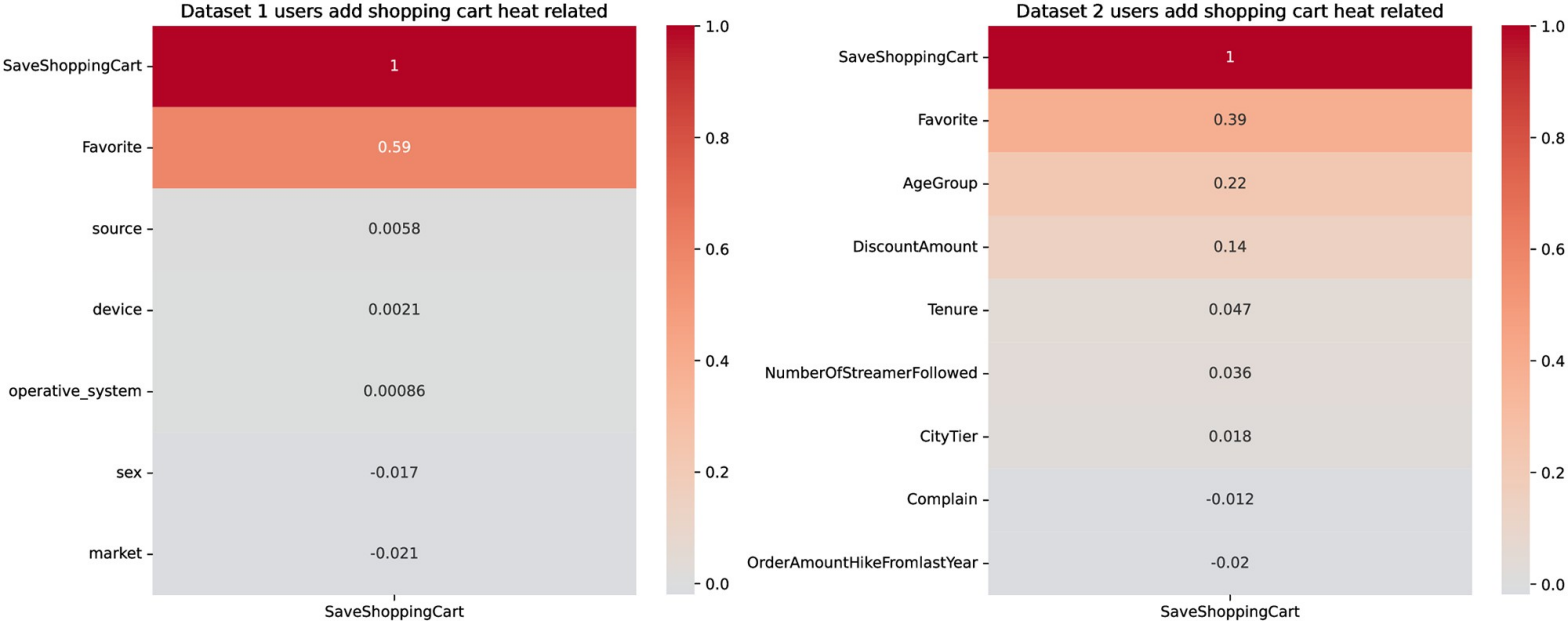
Heatmap related to user adds to the shopping cart.

A heatmap is a data visualization method used to show the correlation or association strength between influencing factors. Typically, heatmaps use colors to indicate the degree of correlation between influencing factors. 0 and 1 in the heatmap represent the degree of correlation between factors, with darker colors typically representing a full positive correlation, denoted by 1. The further away from 1, the less the correlation, usually indicated by lighter colors. Through the heatmap, we explored the relationship between user behaviors related to adding products to the cart and the factors that might influence this action. As shown in Fig 6, we will further analyze the relationship between the top three factors affecting user collection behavior and whether the product was added to the cart.

**Fig 7:**
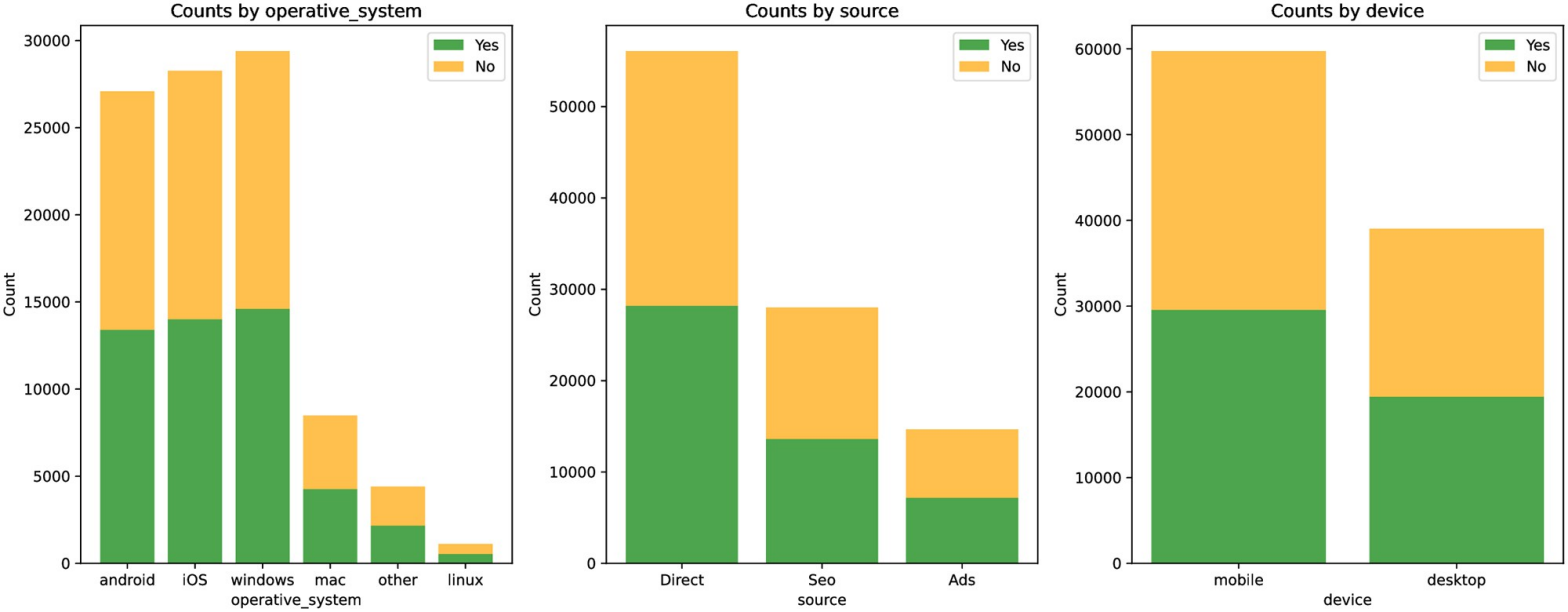
Dataset 1: The relationship between whether a product is added to the shopping cart or not.

Fig 7 details the behavior of users in Dataset 1 when adding products to the cart. Windows users have the highest visit volume, followed by iOS and Android users. Direct product search is the primary source, while search engine and advertisement traffic are slightly lower. With the shift in lifestyle, mobile device visits have surpassed desktop, further emphasizing the importance of a mobile-first strategy.

Table 3 details the behavior of users in Dataset 2 when adding products to the cart. Over time, there are more recent order users among the users who added products to favorites than those who haven’t ordered for a long time. Young users have significantly higher order volumes than older ones, and products with discounts have a much higher order volume than those without discounts.

**Table 3:** Dataset 2: The relationship between whether a product is added to the shopping cart or not.

	Young	Old	DiscountAmount	Not DiscountAmount	Tenure	Not Tenure
Yes	3313	495	3603	95	3545	182
No	1514	308	1914	18	1827	76

**Fig 8:**
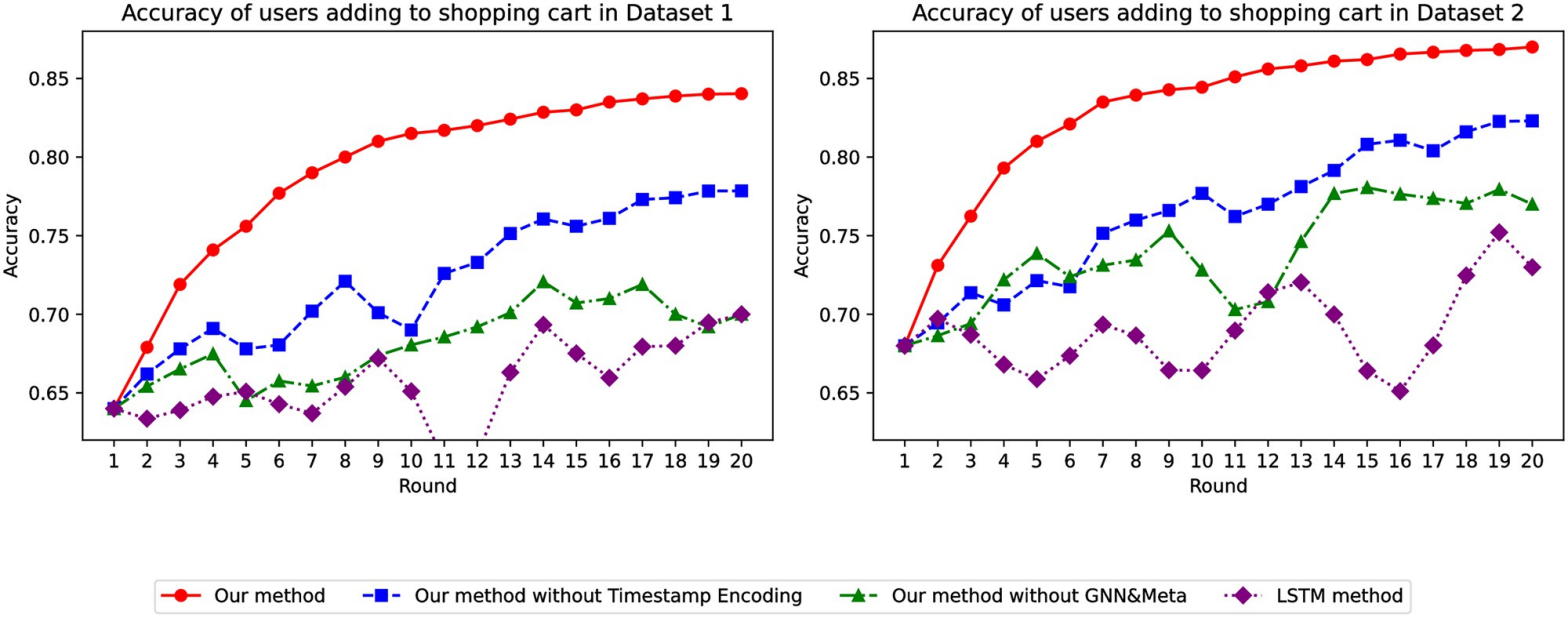
Accuracy of whether the user adds to the shopping cart.

Through the filtering of users who added products to favorites, we experimentally compared the performance between our model and traditional models. As shown in Fig 8, as the global model training epochs increase, our model’s accuracy in predicting whether a user who has favorited a product will add it to the cart has been steadily improving and is even higher than when predicting whether a user would favorite a product. Furthermore, our model has significantly higher accuracy on both Dataset 1 and Dataset 2 compared to when not filtering by favorites. This demonstrates the efficacy of our model in predicting user behavior.

**Fig 9:**
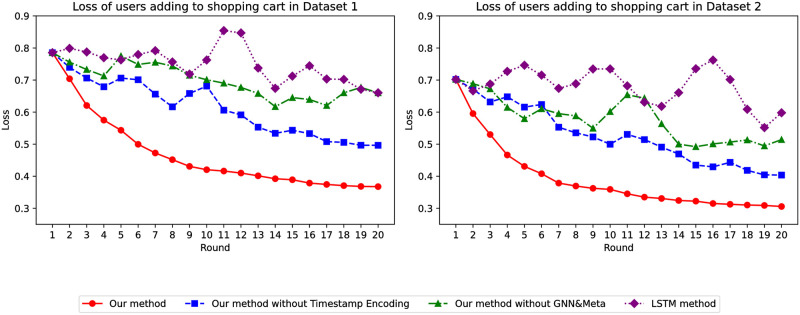
Loss of whether the user adds to the shopping cart.

For loss value, global model training has a certain impact on the stability of our model compared to traditional models. As shown in Fig 9, with the increase in the number of epochs for both datasets, our model’s loss value on both Dataset 1 and Dataset 2 is much better than when not filtering by favorites. Our model consistently decreases in a stable manner, exhibiting relative stability and superiority.

**Fig 10:**
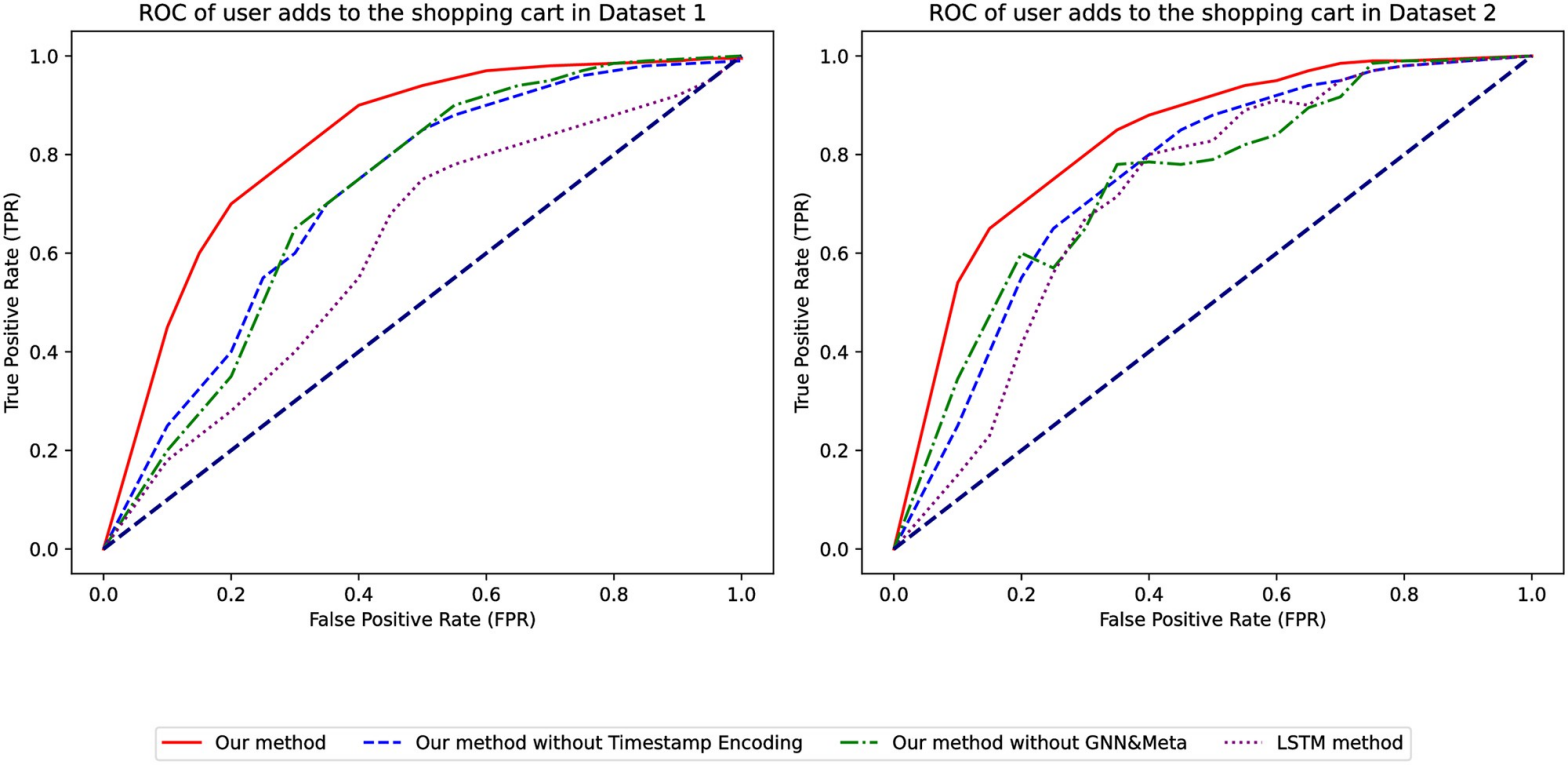
OC of whether the user adds to the shopping cart.

Once again, by comparing the ROC curves of the models, as shown in Fig 10, our model covers the largest area and is above the dashed line in both datasets, indicating its performance is significantly better than random prediction. The area under the ROC curve is larger than when not filtering by favorites, which further demonstrates the superior performance of our model compared to traditional models.

For website operators, they should pay more attention to mobile users, optimize the conversion rate from the listing page to the product page, and consider enhancing search engine optimization and advertising effectiveness. Young users are more active on the platform; the importance of discounted promotional orders is emphasized again by whether the user adds products to the cart. It’s essential to promptly apply our prediction method to predict each user’s behavior and make timely adjustments.

### 5.4 Final decision–purchase phase

Purchasing marks the culmination of the entire shopping journey. When users decide to make a purchase, they might have overcome all their concerns and reservations including those about the price, quality, and delivery policy. At this point, the buying decision is typically based on trust and satisfaction with the product and trust in the platform. Data indicates that users who have saved items to their favorites or added them to their cart are the most likely to buy.

**Fig 11:**
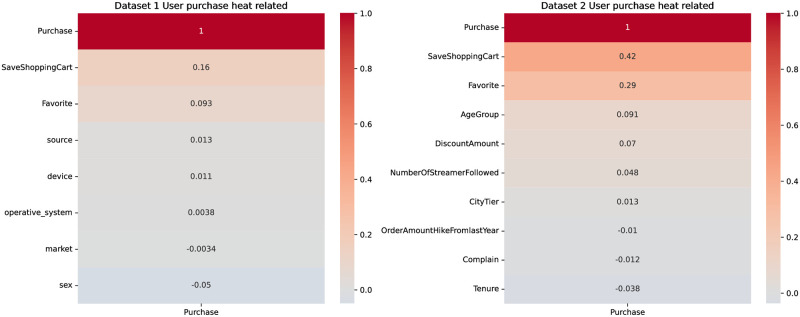
Heat map related to user purchases.

Heatmaps are a data visualization method used to show the correlation or association strength between factors. Typically, heatmaps use colors to depict the degree of correlation between factors, where darker colors represent a perfect positive correlation, often denoted by 1. The further away from 1, the less the correlation, typically shown in lighter colors. We explored the relationship between user buying behavior and the decision to buy, along with factors that might influence this decision using the heatmap, as shown in Fig 11. Next, we will analyze the relationship between the top three factors affecting user’s saving behavior and the decision to buy.

Table 4 provides a detailed display of the buying behavior in Dataset 1. Although Windows users are the most numerous, iOS users have a slightly higher conversion rate. Users from direct sources have the highest number reaching the payment confirmation page. From a conversion rate perspective, those coming directly have the highest conversion rate. Users on mobile devices are more inclined to make a purchase.

**Table 4:** Dataset 1: The purchasing numbers.

Category	Yes	No
Operative System (Android)	621	26470
Operative System (iOS)	684	27579
Operative System (Windows)	677	28715
Operative System (Mac)	324	8156
Operative System (Other)	61	4349
Operative System (Linux)	15	1106
Source (Direct)	1704	54373
Source (SEO)	436	27578
Source (Ads)	242	14424
Device (Mobile)	1364	58362
Device (Desktop)	1018	38013

**Fig 12:**
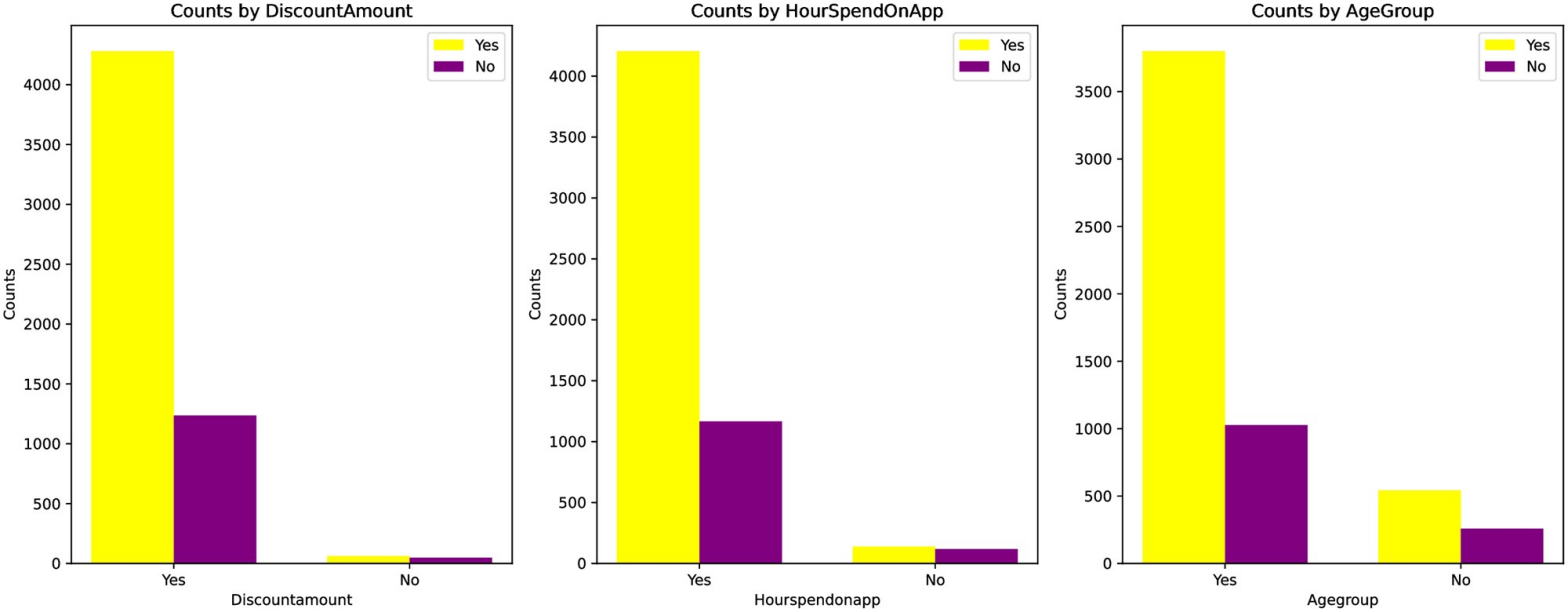
Dataset 2: The relationship between purchasing or not.

Fig 12 details the purchasing behavior in Dataset 2. Users who have already added items to their cart tend to wait for coupons to make a purchase. The longer a user spends on a product, the more likely they are to use a coupon; younger users consistently place more orders than older individuals.

**Fig 13:**
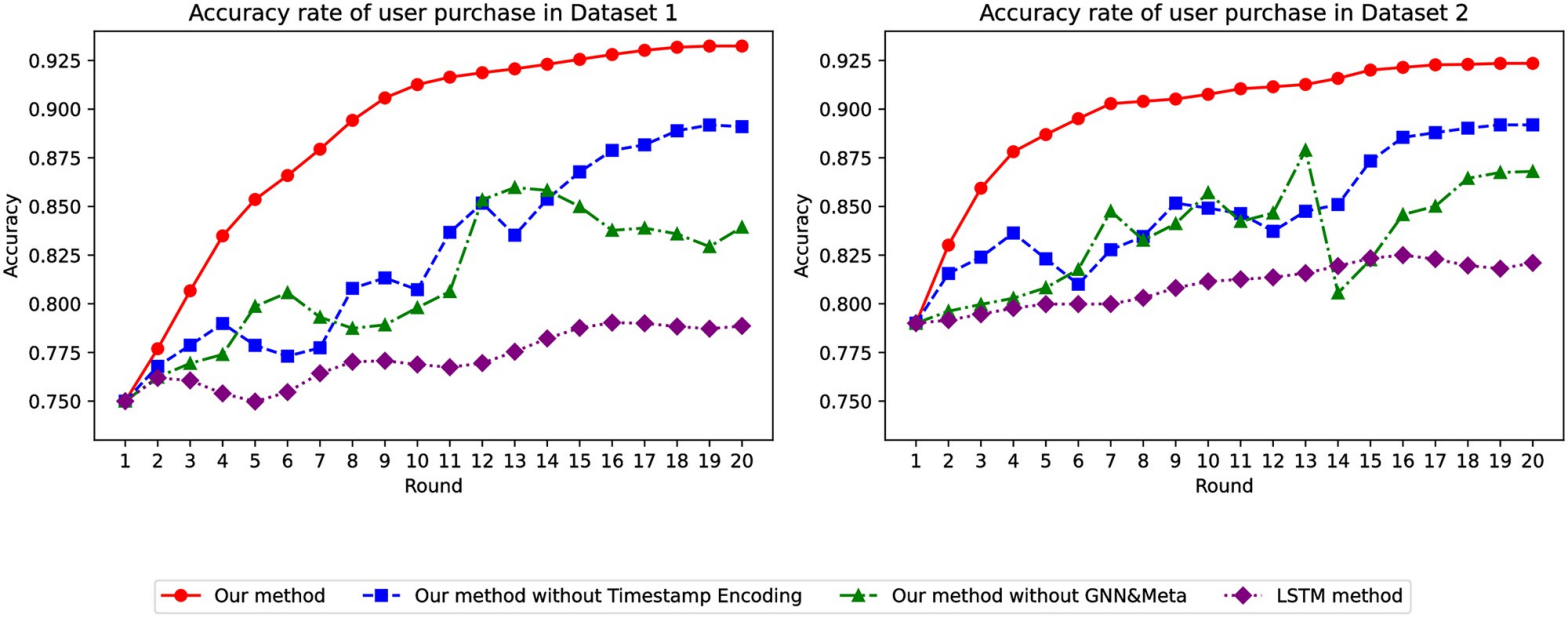
Accuracy of predicting user purchases.

Finally, we compared the performance of various models using data from users who saved items to their favorites and added them to their cart. As Fig 13 indicates, as the global model’s training epochs increased, our model’s accuracy progressively improved. Additionally, our model’s predictions on both Dataset 1 and Dataset 2 significantly outperformed those made before filtering based on user’s saving actions. This confirms the excellent and stable performance of our model, especially in predicting user purchases.

**Fig 14:**
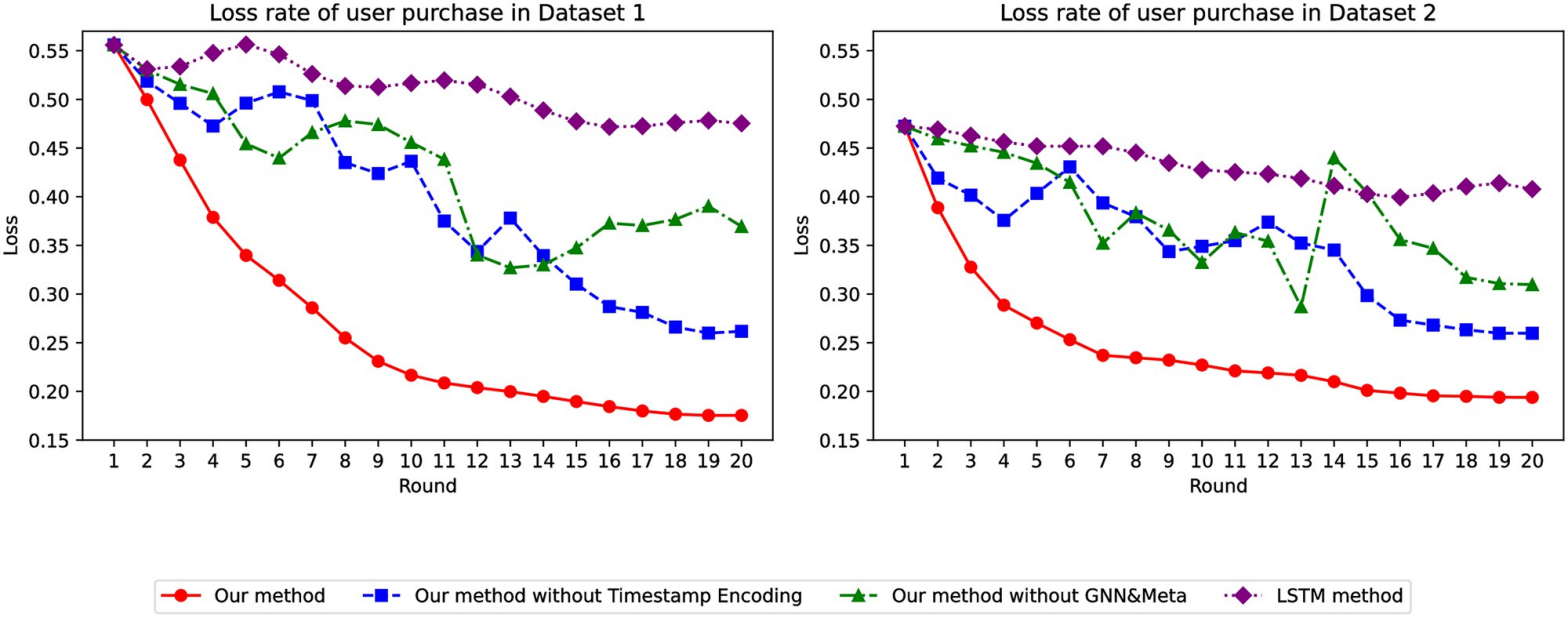
Loss in predicting user purchases.

Regarding the loss value, the global model’s training has certain implications for the stability of our model compared to conventional models. As Fig 14 depicts, compared to the first two behaviors, the loss value is significantly lower, confirming the relative stability and superiority of our model.

**Fig 15:**
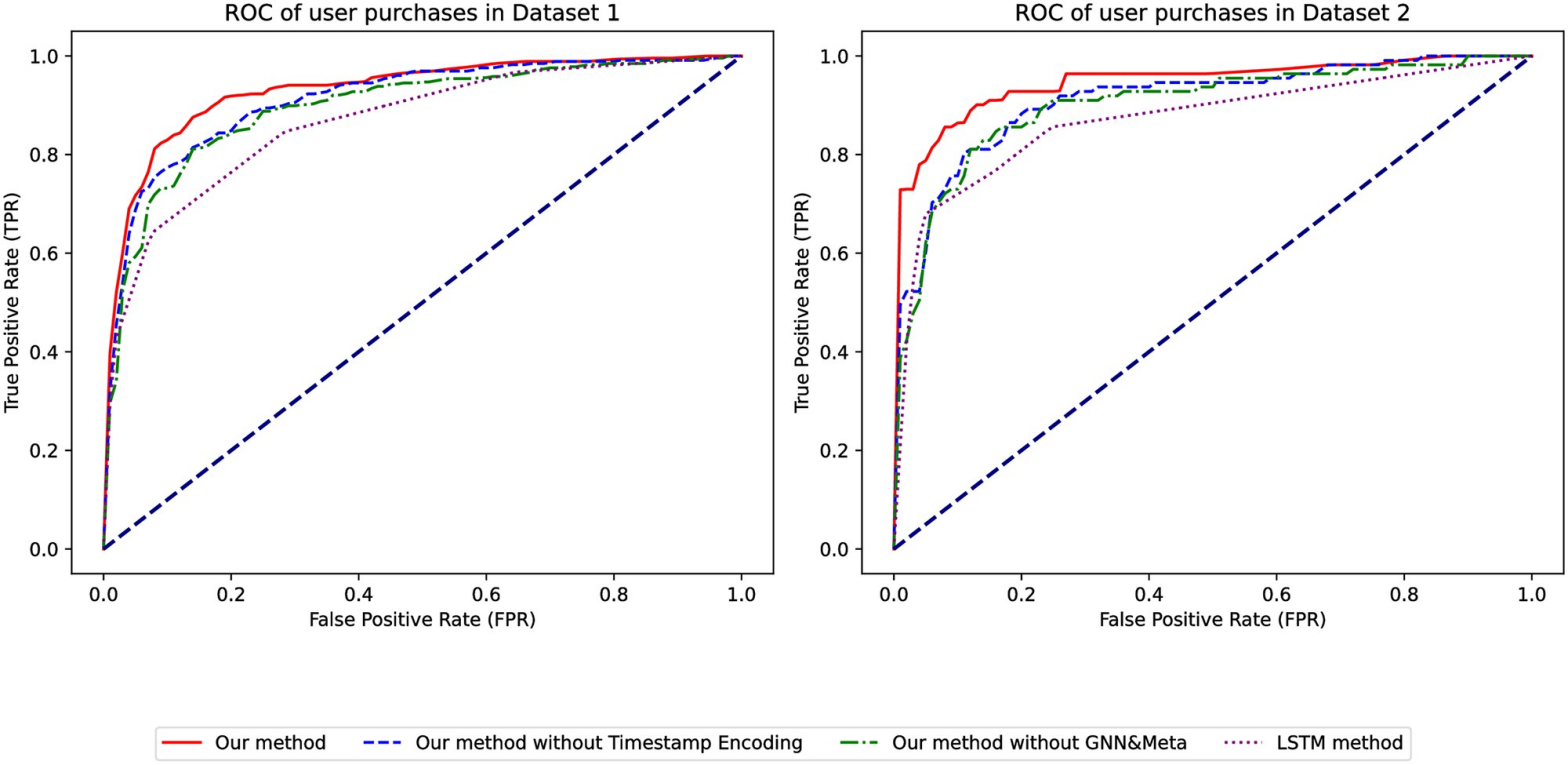
ROC for predicting user purchases.

We further compared the ROC curves of different models, as shown in Fig 15. The area covered by our model’s curve is the largest in both datasets, and it lies above the dashed line, suggesting its performance is significantly better than random predictions. The larger area under the ROC curve confirms the superior performance of our model compared to conventional models.

### 5.5 Discussion

Our investigation into e-commerce user behavior has unveiled several intriguing insights, some of which were unforeseen prior to our experimental analysis. Notably, the distinct impact of targeting specific user demographics, such as iOS users, was more pronounced than initially anticipated. This finding suggests an underexplored potential in segment-specific strategies within e-commerce, a revelation that could steer future marketing tactics.

Furthermore, the effectiveness of personalized incentives, like issuing coupons for products added to carts, revealed a deeper layer of user behavior dynamics. These strategies, while anticipated to be beneficial, yielded results exceeding our initial expectations in their ability to influence purchase decisions. This underscores the hidden complexities in user decision-making processes that are often overshadowed in conventional analysis.

Perhaps most surprisingly, our approach of combining time-series attention with event-based timestamp encoding provided insights beyond traditional purchase behavior predictions. While we expected improvements over standard methods, the degree to which our model captured the temporal nuances of user actions was not fully anticipated. This discovery has significant implications, suggesting that e-commerce platforms may need to reconsider how they interpret and react to user interactions over time.

Reflecting on these findings, we recognize that our research has not only contributed to the advancement of user behavior analysis in e-commerce but also opened new avenues for exploration. The unexpected aspects of our results emphasize the need for continual reassessment of our understanding of user behavior, especially in the ever-evolving landscape of e-commerce.

## 6 Conclusion

Our study provides a comprehensive analysis of the e-commerce shopping journey, elucidating the continuous and logical path users take from item selection to final purchase. The introduction of a time-series attention model integrated with Graph Neural Networks and advanced meta-learning techniques not only enhances the accuracy of purchase behavior predictions but also demonstrates adaptability in rapidly changing e-commerce environments.

Looking forward, our work opens avenues for further research into the intricate relationship between e-commerce user behavior and shopping psychology. Particularly, exploring impulsive buying tendencies in live-streaming environments stands out as a promising future direction. This exploration could provide valuable insights for e-commerce platforms to refine their strategies, thereby increasing user engagement and sales.

The empirical validation of our model through various metrics reinforces its superiority over traditional models, particularly in large-scale and dynamic data scenarios. By addressing the current challenges in e-commerce user behavior analysis, our research lays a robust foundation for future studies to investigate the complex interplay of user behavior, shopping psychology, and technological advancements in e-commerce.

## Supporting information

S1 Appendix Mathematical proof. For the mathematical proof, please refer to the document Appendix_Mathematical_Proof.
(PDF)
