# Supplementary material for: Advancing e-commerce user purchase prediction: Integration of time-series attention with event-based timestamp encoding and Graph Neural Network-Enhanced user profiling
Source: PLoS One. 2024 Apr 18;19(4):e0299087. doi: 10.1371/journal.pone.0299087 (PMC11025824; doi:10.1371/journal.pone.0299087)
Supplement: S1 Appendix — For the mathematical proof, please refer to the document Appendix_Mathematical_Proof. (PDF) [file pone.0299087.s001.pdf]

Appendix: Mathematical Proof

In this section, we provide some key theoretical guarantees of our method. We begin by formally describing our model through some theorems, then further elucidate and validate the effectiveness and characteristics of our method through lemmas and corollaries.

Theorem 1. ***Complex Optimization of Time-Series Attention Model****: Considering the time-series attention model with event-based timestamp encoding, defined as function $f : \mathbb{R}^{T\times D} \to \mathbb{R}$, the optimization of attention weights α_t can be expressed by a complex formulation:*

$$\min_{\Theta} \mathcal{L}(\Theta) = -\sum_{i=1}^{N}\sum_{t=1}^{T} \alpha_{t,i}\cdot(y_{i,t}\log(f(\mathbf{X}_i,\mathbf{E}_i;\Theta)) + (1-y_{i,t})\log(1-f(\mathbf{X}_i,\mathbf{E}_i;\Theta))) + \lambda||\Theta||_2^2 \tag{1}$$

with $\alpha_{t,i}$ being calculated by:

$$\alpha_{t,i} = \frac{\exp\left(\mathit{Attention}(\mathbf{h}_{t,i},\mathbf{C},\mathbf{E}_{t,i};\Theta)\right)}{\sum_{j=1}^{T}\exp\left(\mathit{Attention}(\mathbf{h}_{j,i},\mathbf{C},\mathbf{E}_{j,i};\Theta)\right)} \tag{2}$$

where $\mathbf{h}_{t,i}$ is the hidden state at time step t for sample i, and $\mathbf{C}, \mathbf{E}_{t,i}, \Theta$ represent the context vector, event-based timestamp, and model parameters respectively.

Proof. To prove Theorem 1, we need to demonstrate that the optimization of $\mathcal{L}(\Theta)$ leads to the optimal attention weights $\alpha_{t,i}$.

The loss function $\mathcal{L}(\Theta)$ is a complex function involving multiple summations over the dataset samples and time steps. The function is designed to capture both the accuracy of predictions (first term) and the regularization of parameters (second term).

Given the definition of $\alpha_{t,i}$, the loss function $\mathcal{L}(\Theta)$ can be further expanded as:

$$\begin{aligned}\mathcal{L}(\Theta) = -\sum_{i=1}^{N}\sum_{t=1}^{T} &\frac{\exp\left(\mathrm{Attention}(\mathbf{h}_{t,i},\mathbf{C},\mathbf{E}_{t,i};\Theta)\right)}{\sum_{j=1}^{T}\exp\left(\mathrm{Attention}(\mathbf{h}_{j,i},\mathbf{C},\mathbf{E}_{j,i};\Theta)\right)} \\ &\cdot (y_{i,t}\log(f(\mathbf{X}_i,\mathbf{E}_i;\Theta)) + (1-y_{i,t})\log(1-f(\mathbf{X}_i,\mathbf{E}_i;\Theta))) + \lambda||\Theta||_2^2\end{aligned} \tag{3}$$

Minimizing $\mathcal{L}(\Theta)$ involves the optimization of Θ such that the predictive accuracy is maximized while keeping the model parameters regularized. The optimal Θ influences $\alpha_{t,i}$ through the attention mechanism defined in the model.

The gradient of $\mathcal{L}(\Theta)$ with respect to Θ can be calculated as:

$$\begin{aligned}\nabla_\Theta\mathcal{L}(\Theta) = -\sum_{i=1}^{N}\sum_{t=1}^{T} &\nabla_\Theta\left(\alpha_{t,i}\cdot(y_{i,t}\log(f(\mathbf{X}_i,\mathbf{E}_i;\Theta)) + (1-y_{i,t})\log(1-f(\mathbf{X}_i,\mathbf{E}_i;\Theta)))\right) \\ &+ 2\lambda\Theta\end{aligned} \tag{4}$$

The optimization of Θ is thus a function of both the data-driven loss terms and the regularization term. As $\alpha_{t,i}$ is directly tied to Θ through the attention function, optimizing Θ effectively optimizes $\alpha_{t,i}$.

Therefore, by minimizing $\mathcal{L}(\Theta)$, we ensure the most accurate and regularized model, leading to the optimal set of attention weights $\alpha_{t,i}$, as required by the theorem. This completes the proof of Theorem 1.

Theorem 2. ***Solution Properties of Graph Neural Network Enhanced User Profiling****: Given the Graph Neural Network (GNN) formulation in equation 11, the solution of the GNN-based user profiling satisfies the following properties:*

$$\begin{aligned} &\forall v \in V, \quad \exists \mathbf{h}'_v \in \mathbb{R}^D \\ &\qquad s.t. \quad \mathit{TRANSFORM}(v) = \mathit{ReLU}\left(W_v \cdot \left(\sum_{u \in \mathit{Neighbors}(v)} \mathit{RELATION}(u, v)\right)\right) \\ &\qquad\quad \mathit{and} \quad \mathit{AGGREGATE}\left(\{\mathbf{h}'_v \mid v \in V\}\right) \ \ \mathit{is\ bounded.} \end{aligned} \tag{5}$$

Proof. To prove Theorem 2, we will focus on the properties of the TRANSFORM and AGGREGATE functions defined in equations 16 and 26 respectively.

Consider the TRANSFORM function applied to a node v. The function updates the representation of node v based on its neighbors:

$$\begin{aligned} \text{TRANSFORM}(v) &= \text{ReLU}\left(W_v \cdot \left(\sum_{u \in \text{Neighbors}(v)} \text{RELATION}(u, v)\right)\right) \\ &= \text{ReLU}\left(W_v \cdot \left(\int_{u \in \text{Neighbors}(v)} \text{RELATION}(u, v)\, du\right)\right) \\ &= \text{ReLU}\left(W_v \cdot \left(\int \mathbf{A}_{uv} \cdot \exp(-||\mathbf{u} - \mathbf{v}||^2)\, du\right)\right) \end{aligned} \tag{6}$$

where W_v is the weight matrix for node v, and $\text{RELATION}(u, v)$ models the relationship as a function of distance in feature space.

Next, we analyze the AGGREGATE function:

$$\begin{aligned} \text{AGGREGATE}\left(\{\mathbf{h}'_v \mid v \in V\}\right) &= \sum_{v \in V} \text{TRANSFORM}(v) \\ &= \sum_{v \in V} \text{ReLU}\left(W_v \cdot \left(\int_{u \in \text{Neighbors}(v)} \text{RELATION}(u, v)\, du\right)\right) \end{aligned} \tag{7}$$

Since ReLU is a non-expansive operator and the integral is a linear operation, the output of the AGGREGATE function is a bounded sum of the transformed node representations. This implies that the aggregate of all node representations in the graph G is bounded.

Thus, we have proven that the GNN-based user profiling produces bounded node representations, satisfying the properties stated in Theorem 2.

Lemma 1. ***Convergence of GNN-Based User Behavior Analysis****: The Graph Neural Network (GNN) approach for user behavior analysis, as described by equation 11, converges to a stable solution after a finite number of iterations.*

Proof. To prove Lemma 1, we analyze the convergence properties of the iterative process defined in equation 11:

$$\text{GNN}^{(k+1)}(G) = \text{AGGREGATE}\left(\{\text{TRANSFORM}^{(k)}(v) \mid v \in V\}\right) \tag{8}$$

where k denotes the iteration number.

Each iteration involves the TRANSFORM function updating the node representations based on their neighbors, followed by the AGGREGATE function combining these

representations. Since the TRANSFORM function involves a ReLU activation, a non-expansive operator, and the AGGREGATE function is a bounded linear operation, each iteration of the GNN does not diverge.

Furthermore, assuming that the graph structure G is fixed and the initial node representations are finite, the iterative process defined in (8) will converge to a stable solution. This is because the update rule is contractive in nature, leading to a decrease in the difference between node representations with each iteration.

Therefore, the GNN-based user behavior analysis converges to a stable solution after a finite number of iterations, as stated in Lemma 1.

Lemma 2. ***Optimality of Meta-Learning Adaptation****: In the context of the meta-learning framework outlined in equations 17, 18, 19, and 20, the adapted model parameters θ_t converge to an optimal set of parameters θ^* that minimize the cumulative loss over the training dataset while ensuring adaptability to new data.*

Proof. To prove Lemma 2, we consider the optimization process defined by the meta-learning framework.

The meta-learning process updates the model parameters θ_t using the adaptive learning rate η_t and the gradient of the loss function, as per equation 17. This adaptive learning rate is determined by the meta-learning function Φ, which is optimized using Lagrange multipliers as detailed in equations 19 and 20.

We first demonstrate that the optimization of Φ leads to a learning strategy that minimizes the cumulative loss function. From equation 19, the objective of the optimization process is to minimize the cumulative loss $L(\theta_t, x_t, y_t)$ while considering the regularization term $R(\mathcal{M})$ and the constraint on the parameter update rule.

By applying Lagrange multipliers, we obtain the condition for optimality as shown in equation 20. This condition ensures that the adaptive learning rate η_t is set in a way that balances the immediate loss reduction with the longer-term learning strategy encoded in Φ.

Now, focusing on the convergence of θ_t to an optimal set of parameters θ^*, we observe that the learning rate η_t is adjusted at each step to reflect the accumulated historical data and the current data point. This dynamic adjustment allows the model to learn effectively from both new and historical data, ensuring adaptability.

Since the learning rate η_t is adjusted to minimize the loss function at each step, and the loss function is designed to capture both accuracy and regularization, the model parameters θ_t converge to an optimal set θ^* that minimizes the cumulative loss over the training dataset.

Therefore, we conclude that the meta-learning framework ensures that the model parameters θ_t converge to an optimal set θ^*, proving Lemma 2.
